# Supplementary material for: AffyMAPSDetector: a software tool to characterize Affymetrix GeneChip™ expression arrays with respect to SNPs
Source: BMC Bioinformatics. 2007 Jul 30;8:276. doi: 10.1186/1471-2105-8-276 (PMC1959249; doi:10.1186/1471-2105-8-276)
Supplement: Additional file 13 — AffyMAPSDetector v1 source code. AffyMAPSDetector source code. [file 1471-2105-8-276-S13.zip › AffyMAPSDetector_v1_src/doc/index-files/index-3.html]

C-Index


|  |  |  |  |
| --- | --- | --- | --- |
| **Overview** \| Package \| Class \| Use \| **Tree** \| **Deprecated** \| **Index** \| **Help** | | |  |
| **PREV LETTER**   **NEXT LETTER** | **FRAMES**    **NO FRAMES**     **All Classes** |  |  |


A B C D E F G H I J L M N O P R S T V W X Y 

---


## **C**

**CANCEL\_I18N** - Static variable in class jwf.Wizard: **ColorGradient** - class affychip.snpdetector.util.ColorGradient.: Title: Probe SNP Detector **ColorGradient(Color, Color)** - Constructor for class affychip.snpdetector.util.ColorGradient: **ComplementDnaSeq** - class affychip.snpdetector.util.ComplementDnaSeq.: Title: Probe SNP Detector **ComplementDnaSeq()** - Constructor for class affychip.snpdetector.util.ComplementDnaSeq: **Constant** - class affychip.snpdetector.Constant.: Title: Probe SNP Detector **Constant()** - Constructor for class affychip.snpdetector.Constant: **CrossHbCheckFrame** - class affychip.snpdetector.ui.CrossHbCheckFrame.: Title: Probe SNP Detector **CrossHbCheckFrame(JFrame)** - Constructor for class affychip.snpdetector.ui.CrossHbCheckFrame: **canFinish()** - Method in class affychip.snpdetector.ui.StepOnePanel: Can this panel finish the wizard? **canFinish()** - Method in class affychip.snpdetector.ui.StepThreePanel: **canFinish()** - Method in class affychip.snpdetector.ui.StepTwoPanel: **canFinish()** - Method in class jwf.NullWizardPanel: Can this panel finish the wizard? **canFinish()** - Method in class jwf.WizardPanel: Can this panel finish the wizard? **clearState()** - Method in class affychip.snpdetector.model.DataState: **columns** - Variable in class affychip.snpdetector.util.PmMmHeatmapViewerModel: **compare(Object, Object)** - Method in class affychip.snpdetector.util.PmMmHeatmapViewerModel.RowComparatorForDataSorting: **compareTo(Object)** - Method in class affychip.snpdetector.model.Probe: **compareTo(Object)** - Method in class affychip.snpdetector.model.ProbeSet: **compareTo(Object)** - Method in class affychip.snpdetector.util.PmMmHeatmapViewerModel.Row: **createImageIcon(String)** - Static method in class affychip.snpdetector.resources.AppIcons: **createTable()** - Method in class affychip.snpdetector.ui.AffyMapsDetectorFrame

---


|  |  |  |  |
| --- | --- | --- | --- |
| **Overview** \| Package \| Class \| Use \| **Tree** \| **Deprecated** \| **Index** \| **Help** | | |  |
| **PREV LETTER**   **NEXT LETTER** | **FRAMES**    **NO FRAMES**     **All Classes** |  |  |


A B C D E F G H I J L M N O P R S T V W X Y 

---
